# Two IIIf Clade-bHLHs from *Freesia hybrida* Play Divergent Roles in Flavonoid Biosynthesis and Trichome Formation when Ectopically Expressed in *Arabidopsis*

**DOI:** 10.1038/srep30514

**Published:** 2016-07-28

**Authors:** Yueqing Li, Xiaotong Shan, Ruifang Gao, Song Yang, Shucai Wang, Xiang Gao, Li Wang

**Affiliations:** 1 Key Laboratory of Molecular Epigenetics of MOE, Changchun, China; 2 Institute of Genetics and Cytology, Northeast Normal University, Changchun, China

## Abstract

The MBW complex, comprised by R2R3-MYB, basic helix-loop-helix (bHLH) and WD40, is a single regulatory protein complex that drives the evolution of multiple traits such as flavonoid biosynthesis and epidermal cell differentiation in plants. In this study, two IIIf Clade-bHLH regulator genes, *FhGL3L* and *FhTT8L*, were isolated and functionally characterized from *Freesia hybrida*. Different spatio-temporal transcription patterns were observed showing diverse correlation with anthocyanin and proanthocyanidin accumulation. When overexpressed in *Arabidopsis*, *FhGL3L* could enhance the anthocyanin accumulation through up-regulating endogenous regulators and late structural genes. Unexpectedly, trichome formation was inhibited associating with the down-regulation of *AtGL2*. Comparably, only the accumulation of anthocyanins and proanthocyanidins was strengthened in FhTT8L transgenic lines. Furthermore, transient expression assays demonstrated that FhGL3L interacted with AtPAP1, AtTT2 and AtGL1, while FhTT8L only showed interaction with AtPAP1 and AtTT2. In addition, similar activation of the *AtDFR* promoter was found between AtPAP1-FhGL3L/FhTT8L and AtPAP1- AtGL3/AtTT8 combinations. When FhGL3L was fused with a strong activation domain VP16, it could activate the *AtGL2* promoter when co-transfected with AtGL1. Therefore, it can be concluded that the functionality of bHLH factors may have diverged, and a sophisticated interaction and hierarchical network might exist in the regulation of flavonoid biosynthesis and trichome formation.

Flavonoids and trichomes are specialized metabolites and structures in plants, respectively. The early flavonoid biosynthetic steps are even discovered in the bryophytes (*Physcomitrella patens* and *Marchantia polymorpha*), implying that flavonoids might have evolved to act firstly as chemical messengers and then UV sunscreens^1^. With the evolution of the land plants, the flavonol-to-anthocyanin ratio changes upon the transition to flowering as well as the formation of trichomes, which is considered to be a recent evolutionary trait having evolved from the duplication and diversification of anthocyanin-controlling genes^2^,^3^. It has been hypothesized that the acropetal accumulation of anthocyanins and formation of trichomes on stems contribute to coordinated defenses against crawling herbivores. Anthocyanins provide visual cues that distract herbivores, while trichomes endow plants with physical protection against herbivores^4^.

A major objective of evolutionary developmental biology (‘EvoDevo’) is to elucidate the genetic bases that result in the evolution of novel characters^5^. In the past several decades, the regulatory mechanism involved in the flavonoid biosynthesis and the trichome formation has been well characterized in land plants, three distinct transcription factor gene families, containing R2R3- MYB, basic helix–loop–helix (bHLH) and WD40 repeats (WDRs), comprise an evolutionarily conserved single regulatory protein complex (designated as MBW complex), driving the evolution of multiple traits such as (pro) anthocyanin and anthocyanin biosynthesis and epidermal cell differentiation in plants^6^,^7^,^8^. MBW partners have also been well characterized in *Arabidopsis*, *Maize*, *Petunia*, *Tobacco* and other angiosperms, especially in dicots^6^,^8^,^9^.

So far, the most extensively studied MBW complex has been elucidated in *Arabidopsis* (*Arabidopsis thaliana*), which has been implicated in five traits: anthocyanin and proanthocyanidin biosynthesis, seed coat mucilage production, and trichome and root hair patterning. In the complex, three bHLH proteins, i.e. *AtTT8*, *AtGL3* and *AtEGL3*, regulate these five traits in a partially redundant manner, whereas the R2R3 MYB factors regulate individual traits^9^,^10^,^11^,^12^. The bHLH proteins are a large class of transcription factors in plants, and have been divided into 26 subgroups^13^. In *Arabidopsis*, 12 subfamilies have been identified and the aforementioned three bHLHs have been grouped into subgroup IIIf, all of which have been proved to be involved in both flavonoid biosynthesis and trichome formation^14^. As for the flavonoid biosynthesis, AtTT8 interacts with specialized MYBs, AtPAP1, AtPAP2, AtMYB113 and AtMYB114 to control the spatio-temporal anthocyanin accumulation. Furthermore, AtGL3 and AtEGL3 also fulfill the roles in the anthocyanin biosynthesis when equipped with MYB regulators^9^,^15^. In the proanthocyanidin biosynthesis process, the AtTT2–AtTT8–AtTTG1 complex plays the major role in developing seeds, and three additional MBW complexes (i.e. AtMYB5–AtTT8–AtTTG1, AtTT2–AtEGL3–AtTTG1 and AtTT2–AtGL3–AtTTG1) were also shown to be involved in a tissue specific manner^16^,^17^. In addition, a hierarchical relationship between *AtGL3*, *AtEGL3* and *AtTT8* has been observed, both *AtGL3* and *AtEGL3* can additively contribute to the regulation of *AtTT8*, and *AtEGL3* contributes more to *AtTT8* regulation than *AtGL3*^15^. In regard to trichome formation, *AtGL3* and *AtEGL3* functions partial redundantly for trichomes formation on both leaf lamina and stem^3^,^12^, and a recent report also showed that *AtTT8* was involved in the development of marginal trichomes of rosette leaves treated with jasmonic acid, 6-benzylaminopurine and gibberellic acid^18^. The R2R3 MYB gene acting specifically in trichome patterning is represented by *AtGL1*, and the AtGL1-AtGL3-AtTTG1 complex transcriptionally activates the homeobox transcription factor GLABRA2 (GL2) and other downstream genes, which in turn promotes the trichomes differentiation^10^,^12^. Interestingly, in our previous studies, we found that AtGL2 negatively regulated anthocyanin biosynthesis in *Arabidopsis* via directly repressing the expression of some MBW component genes^19^.

To date, some common TF components of the MBW complex have been found in angiosperms as well as gymnosperms and mosses^20^,^21^, and the bHLH-interacting domain located in these R2R3-MYB proteins is well conserved among higher plant species. Therefore, it is reasonable to deduce that at least MYB interactions and probably MBW complex arose early during land plant evolution. After the division of monocots and dicots, it might function divergently in flavonoid biosynthesis suffered from diverse positive selection pressures. In the monocot maize, the anthocyanin biosynthesis genes are activated as a single unit by a ternary MBW complex. In the dicot *Arabidopsis*, anthocyanin biosynthesis genes can be divided into two subgroups: early biosynthesis genes (EBGs) are activated by co-activator independent R2R3-MYB transcription factors, whereas late biosynthesis genes (LBGs) require an MBW complex. Among the MBW complex members, MYB proteins are the key components providing specificity for the subsets of genes activated, which have been widely investigated and identified in crop, ornamental, and model plants^22^,^23^. Comparably, the roles of bHLH regulators in MBW complex are less clear, especially in monocotyledons plants. To our knowledge, no divergent IIIf bHLH regulators from a single monocot plant have been reported participating in both flavonoid synthesis and trichome formation yet except maize, although several transcription factors have been characterized in lilies^24^,^25^,^26^, and orchids^27^,^28^.

*Freesia*, a monocotyledonous genus in the Iridaceae consisting of 15 species, originates in southern Africa. It has the potential to be one of the classic model systems for investigating flavonoid biosynthesis and regulation in monocots, particularly for the flower pigmentation. Firstly, the flower has complex floral pigmentation patterns, including petal limb, pistil, anthers and vein associated patterning. Secondly, five kinds of anthocyanin aglycons, i. e. delphinidin, petunidin, malvinidin, peonidin, and cyanidin, were observed as well as two kinds of flavonols, i. e. kaempferol and quercetin derivatives. And the accumulation profile for anthocyanins was the opposite of that for flavonols during the flower development process^29^,^30^. In addition, proanthocyanidins were also found in flowers, indicating a sophisticated transcription regulation network for the flavonoid biosynthesis in the *Freesia* flowers. In our previous studies, two anthocyanin biosynthetic genes, *Fh3GT1* and *FhCHS1* were isolated and functionally verified^29^,^30^,^31^.

In this study, two IIIf Clade-bHLH regulator genes, tentatively designated as *FhGL3L* and *FhTT8L*, were isolated from *Freesia hybrida*. After introduced in to *Arabidopsis*, divergent functionalities were observed in flavonoid biosynthesis and trichome formation, and a model that integrates the interactions of the MYB and bHLH partners within the MBW complex to explain their roles in *Arabidopsis* were proposed. This is, to our knowledge, the first report of the identification of regulatory genes in *Freesia* flowers, and the results will provide new insights into the flavonoid biosynthesis and trichome formation regulation in monocot plants.

## Results

### Identification and sequence analysis of two candidate flavonoid biosynthesis and trichome formation related bHLH transcription factors

**Figure 1:**
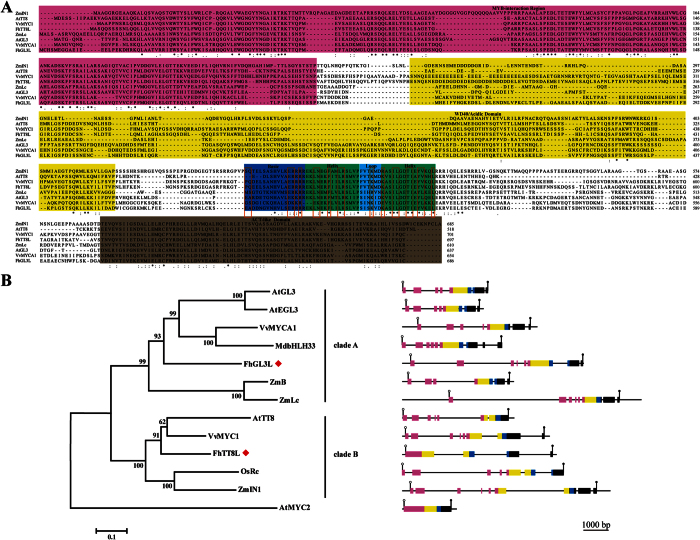
Molecular Analysis of the FhGL3L and FhTT8L Gene. (**A**) Full-length Protein Sequence Alignment of FhbHLHs and Their Closest Homologs in the Regulation of Flavonoid Biosynthesis. Numbers indicated the position of the last amino acid in each line of the proteins within the corresponding full-length protein sequence. *identical amino acids; : or ·, similar amino acids. The MIR (MYB-Interacting Region), the acidic (WD40/AD), the bHLH and ACT-like domains were shaded in different colors. The 19 conserved residues characteristic of the bHLH domain were highlighted using red boxes. (**B**) Phylogenetic Tree and Genome structures of Different bHLH Proteins in Plants. Phylogenetic tree was constructed using the neighbor-joining method by the MEGA6 software. The reliability of the trees was tested using a bootstrapping method with 1000 replicates. Numbers indicated bootstrap values for 1000 replicates. FhbHLHs were indicated with red boxes. Both exons and introns were drawn to scale. Coding sequences were indicated by double height. The regions encoding the conserved MIR domain, acidic domain (WD40/AD), bHLH domain and C-terminal domain were indicated by reddish purple, yellow, blue and black boxes, respectively. The open and closed circles denoted the start and stop codons, respectively, of the protein coding region. The GenBank accession numbers of the bHLH protein sequences were as follows: *Vitis vinifera* VvMYC1 (EU447172), VvMYCA1 (EF193002); *Oryza sativa* OsRc (BAF42667); *Zea mays* ZmB (CAA40544), ZmLc (AAA33504), ZmIN1 (AAB03841); *Malus domestica* MdbHLH33 (ABB84474); *Arabidopsis thaliana* AtTT8 (Q9FT81), AtEGL3 (Q9CAD0), AtGL3 (NP_680372), AtMYC2 (AAM19778.1).

Two putative members of the bHLH family of transcription factors were identified from the transcriptomic database of *Freesia hybrida*, denominated as *FhGL3L* and *FhTT8L*. FhGL3L has an open reading frame (ORF) of 2440 bp encoding 686 amino acids. The 2394 bp FhTT8L encodes 697 amino acids (Table S1). Sequence analysis showed that the MYB interaction region (MIR) was present at the N-terminal region of the proteins; the bHLH domain involved in the formation of homo- or heterodimers with other bHLH proteins or DNA binding was located in C-terminal region. In addition, a sequence rich in acidic amino acids was present between the MIR and bHLH domain, which was believed to interact with WD40 proteins and/or with the RNA polymerase II (Fig. 1A). Phylogenetic analysis suggested that they phylogenetically clustered with two clades (Fig. 1B): clade A and clade B, respectively, as described in Davies *et al*.^32^. Clade A contained GL3/EGL3 (*Arabidopsis*), B, LC (maize), MYCA1 (*Vitis*) and bHLH33 *(Malus)*, whereas clade B included TT8 (*Arabidopsis*), IN1 (maize), MYC1 (*Vitis*) and Rc (rice). In order to obtain the genomic sequences of the two bHLH genes, specific primers (shown in Table S2) were designed and amplified using DNA as template, *FhGL3L* had seven introns, whereas *FhTT8L* had three introns, all the introns began with the nucleotides GT and ended with the nucleotides AG, following the GT-AG rule (Fig. 1B). In addition, genomic DNA sequences from monocots had more similar structures, indicating a more close relationship during evolution.

### Transactivation Properties of FhGL3L and FhTT8L

**Figure 2:**
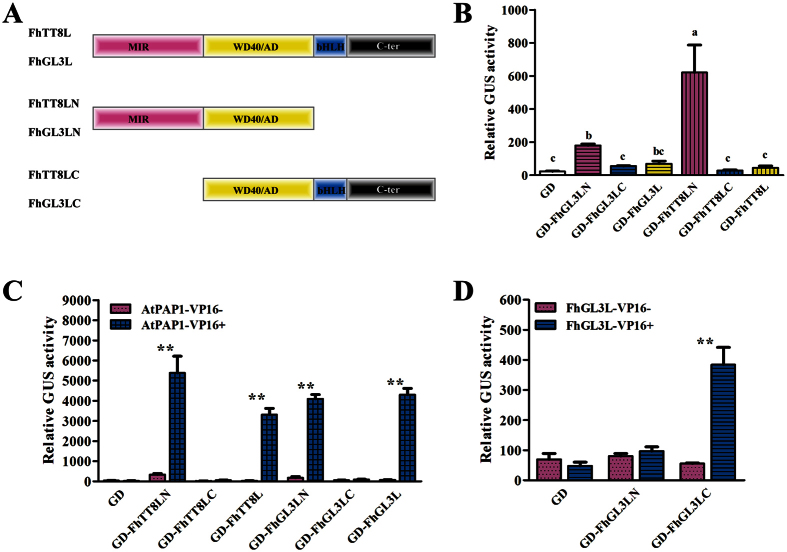
Function of Different Regions of FhGL3L and FhTT8L. (**A**) Schematic diagram of the full-length and partial truncated FhbHLHs. (**B**) Transactivation capacity of different domains of FhbHLHs. (**C**) Different truncated FhbHLH peptides showed different interaction capacity with AtPAP1. The N-terminal region was indispensable for the interaction with MYB partners. (**D**) The C-terminal region of FhGL3L was indispensable in dimerization. Activation of GAL4 by the respective constructs was determined by measuring beta-glucuronidase activity. The plasmids were co-transfected into protoplasts isolated from *Arabidopsis* rosette leaves. Protoplasts were incubated in darkness for 20–22 h after transfection, and then GUS activity was measured. Data represented the mean ± SD of three replicates. Constructs were diagrammed at the bottom of the figure. One-way ANOVA was carried out to compare statistical differences in Fig. 5B. (Ducan, p < 0.05). T-test was used to analysis the significant defference in Fig. 5C,D (*P < 0.05; **p < 0.01). All tests were computed using SPSS(ver.17.0).

To determine the transactivation properties of the FhGL3L and FhTT8L proteins and investigate the roles of different regions in the proteins, three kinds of vectors harboring full-length and partially truncated FhbHLHs, designated as FhGL3L/FhTT8L, FhGL3LN/FhTT8LN (lacking C-terminus) and FhGL3LC/FhTT8LC (lacking N-terminus), respectively (Fig. 2A), were fused to GD tagged constructs and transiently introduced into *Arabidopsis* leaf protoplasts. The reporter plasmid Gal4-GUS was cotransformed with GD-tagged genes. Protoplasts transfected with *FhGL3L* or *FhTT8L* effector constructs exhibited a very weak beta-glucuronidase (GUS) activity same as the negative control, indicating that both bHLH proteins lack transactivation capacity. However, when the C-terminus region was truncated, both FhGL3LN and FhTT8LN were able to promote the GUS activity despite the transactivation ability of FhGL3LN was not significant different compared with full length protein (Fig. 2B), implying that the WD40/AD domain was necessary for the transactivation capacity, while the C-terminus region performed counteraction roles. In order to verify the function of different regions of FhGL3L and FhTT8L in interaction with MYB partners, all the full-length and partially truncated FhbHLHs aforementioned were co-transfected with AtPAP1-VP16. As shown in Fig. 2C, no interaction was found between AtPAP1 and proteins lacking N-terminal region (FhGL3LC/FhTT8LC). In addition, FhGL3LN and FhGL3LC were also used to verify the region involving in dimerization. Only GD-tagged FhGL3LC could highly induce GUS activity when co-transfected with HA-FhGL3L-VP16 (Fig. 2D). These results strongly supported the conclusion that the N-terminal region was indispensable for the transactivation ability and the interaction with MYB partners, while the C-terminal region played important roles in dimerization^22^,^33^,^34^,^35^.

### The expression of *FhTT8L* and *FhGL3L* showed different correlations with flavonoid accumulation in flower developmental process and plant tissues

**Figure 3:**
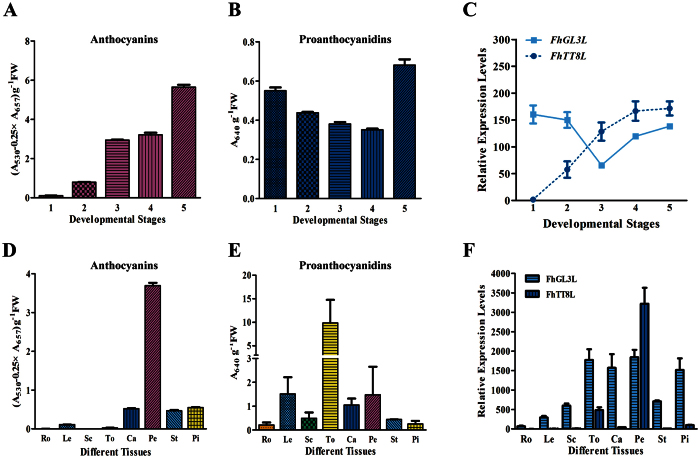
Gene Expression Profiles of *FhGL3L* and *FhTT8L* in *Freesia hybrida.* (**A,D**) The anthocyanin accmulation at different developmental stages and in different tissues. (**B,E**) The proanthocyanidin accmulation at different developmental stages and in different tissues. (**C,F**) Expression profile of *FhGL3L* and *FhTT8L* in flowers at different developmental stages and in different tissues. Data represent means ± SD of three biological replicates. 1–5, represented the flowers of different developmental stages as described earlier^29^,^30^, Ro, roots; Le, leaves; Sc, scapes; To, toruses; Ca, calyxes; Pe, petals; St, stamens; Pi, pistils. FW, fresh weight.

The transcription of two FhbHLHs was compared with the flavonoid accumulation pattern during flower developmental process, which was divided into five stages as described in our previous studies^29^,^30^. In agreement with the flower pigmentation appearance, the amount of anthocyanins increased gradually and peaked at stage 5 when flowers fully opened (Fig. 3A), whereas the proanthocyanidins were constitutively accumulated at a relative lower level, showing no clear correlation with flower coloration (Fig. 3B). Different patterns and levels of *FhGL3L* and *FhTT8L* were observed among the five flower development stages, *FhTT8L* showed an expression pattern synchronous to the anthocyanin accumulation, whereas the expression of *FhGL3L* was not parallel to the flower pigmentation process, the transcripts of which was relative lower in contrast to *FhTT8L* (Fig. 3C) in the late flower development process. Therefore, it can be concluded that *FhTT8L* might fulfill pivotal roles in the anthocyanin biosynthesis during the flower pigmentation. However, because *FhGL3L* presented an early high expression pattern, it might have an indispensable effect on the pigmentation initiation.

In order to study the patterns of transcript accumulation for the two FhbHLHs and their correlation to flavonoids accumulation in plant tissues, 3 vegetative tissues, i.e. root, leaf and scape, and 5 flower tissues, i.e. torus, calyx, petal, stamen and pistil, were collected as described previously^29^,^30^. As shown in Fig. 3D,E, the contents of anthocyanins and proanthocyanidins varied among different tissues. Briefly, flower tissues accumulated higher levels of both two kinds of flavonoid than vegetative tissues; petal and torus was the dominant tissue for anthocyanin and proanthocyanidin biosynthesis, respectively. Comparable levels of *FhGL3L* and *FhTT8L* expressions were observed in the flavonoid accumulated tissues, implying that both bHLH regulators might have an analogous role to control the metabolites biosynthesis in plant tissues. However, it is not always clear if their functions are partially or completely redundant given that the expression level of *FhGL3L* was higher than *FhTT8L* in all the tested tissues except petals (Fig. 3F).

### *FhGL3L* and *FhTT8L* functioned divergently in flavonoid biosynthesis and trichome formation when introduced into *Arabidopsis*

**Figure 4:**
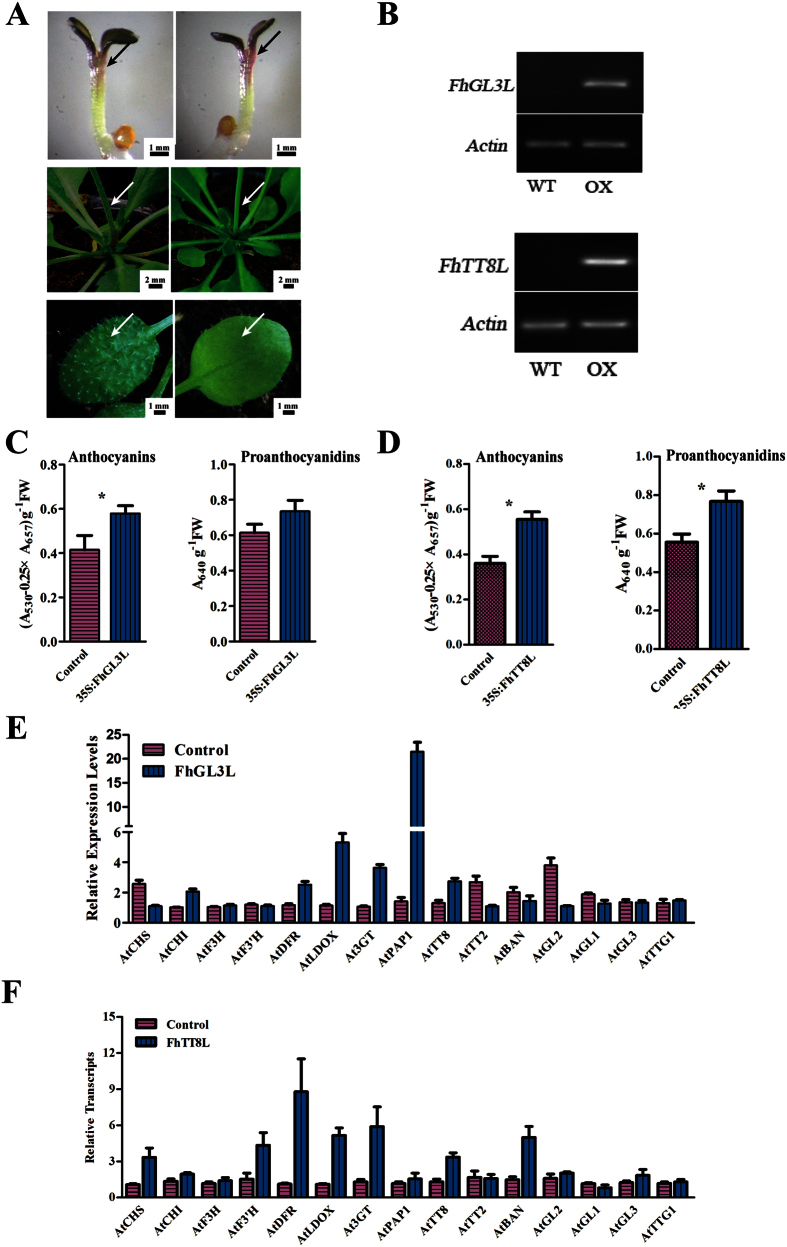
FhGL3L Regulated Anthocyanin Accumulation and Trichome Development while FhTT8L Regulated Anthocyanin and Proanthocyanidin Biosynthetic Genes. (**A**) Phenotypes of wild-type and transgenic *Arabidopsis* seedlings and leaves. (**B**) Expressional analysis of the *FhGL3L* and *FhTT8L* gene by reverse transcription polymerase chain reaction in the wild type and transgenic lines. (**C,D**) Contents of anthocyanins and proanthocyanidins in *Arabidopsis* seedlings in the wild type and *FhGL3L* and *FhTT8L* transgenic lines. (**E,F**) Expressional analysis of genes involved in flavonoid biosynthesis and trichome formation in the wild type, *FhGL3L* and *FhTT8L* transgenic lines by qRT-PCR. Data represented the mean ± SD of three replicates. T-test was used to analysis the significant defference (*P < 0.05; **p < 0.01). All tests were computed using SPSS(ver.17.0).

To ascertain the putative functions of *FhGL3L* and *FhTT8L*, they were introduced into *Arabidopsis* under the control of cauliflower mosaic virus *35S* promoter. The cotyledons and hypocotyls of the seedlings overexpressed *FhGL3L* showed an intense pigmentation phenotype (Fig. 4A). The transgenic lines were confirmed for the presence and expression of exogenous genes through qRT-PCR (Fig. 4B). No amplicon was observed in WT plants whereas amplicon of expected size was observed in transgenic lines expressing *FhGL3L* or *FhTT8L*. The intense pigmentation phenotype in the seedlings overexpressed *FhGL3L* could be represented by a significant enhancement of anthocyanin accumulation (Fig. 4C). Unexpectedly, trichome formation on both leaf lamina and stem trichomes was inhibited (Fig. 4A). Comparably, transgenic lines harboring *FhTT8L* gene showed no significant phenotype differences in trichome formation compared to wild-type lines, however, the accumulation of both anthocyanin and proanthocyanidins was found to be strengthened (Fig. 4D).

In order to identify the putative downstream genes that may be regulated by *FhGL3L*, genes encoding enzymes and transcription factors responsible for the flavonoid biosynthesis were examined by Real-time PCR analysis in seedlings of homozygous transgenic lines (Fig. 4E). Both endogenous LBGs, including *AtDFR*, *AtLDOX* and *At3GT*, and anthocyanin related transcription factors, e.g. *AtPAP1* and *AtTT8*, were coordinately up-regulated, especially for *AtPAP1*, whose expression level was increased over 20 times. The transcript levels of genes participating in trichome formation, such as *AtGL2*, *AtGL1*, *AtGL3* and *AtTTG1*, were also measured. In accordance with trichomes defect phenotype in the transgenic lines, *AtGL2* was significantly down-regulated. Because the transcription patterns of *FhTT8L* were spatially and temporally consistent with anthocyanin and proanthocyanidin accumulation in flower developmental process and different plant tissues, the expression levels of endogenous genes were also examined in *FhTT8L* transgenic line*s*. Real-time PCR results indicated that the LBGs as well as *BAN*, which was specific for the proanthocyandins biosynthesis, were slightly up-regulated when *FhTT8L* overexpressed (Fig. 4F), which was consistent with the results of Arabidopsis leaf protoplasts transient transformation analysis (Figure S1). In conclusion, *FhGL3L* and *FhTT8L* played divergent roles in flavonoid biosynthesis and trichome formation, *FhGL3L* positively regulated anthocyanin accumulation and negatively regulated the formation of trichomes, whereas *FhTT8L* might synchronously control the anthocyanin and proanthocyanidin biosynthesis in combination with the endogenous MYB regulators.

### FhGL3L and FhTT8L interacted with *Arabidopsis* endogenous MYB regulators in distinct ways

**Figure 5:**
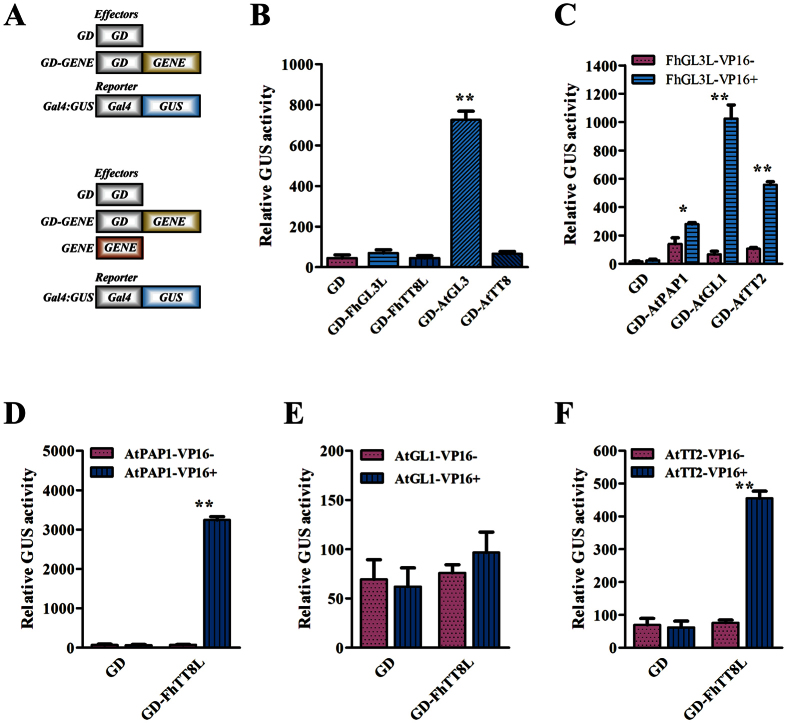
FhGL3L and FhTT8L Physically Interacted with Different *Arabidopsis* MYBs in Plant Cells. (**A**) Schematic diagram of *Arabidopsis* protoplasts transfection assays. Relative GUS activities in *Arabidopsis* mesophyll protoplasts co-transfected with a GUS reporter gene and constructs expressing GAL4 DNA-binding domain (GD) fusion were shown in the top program. Expression of GUS gene occurred if the gene fused to GD had the activating ability. The bottom diagram were the constructs used in detecting the interaction between two proteins. A protein with no or low activation ability was fused to GD. Activation of report only occurred when another protein with activating ability interacted with the GD fusing protein. (**B**) Activation abilities of bHLHs. Only AtGL3 showed high activating ability while others not. (**C**) FhGL3L physically interact with AtPAP1, AtGL1 and AtTT2. (**D–F**) FhTT8L physically interacted with AtPAP1 and AtTT2 but not with AtGL1. Activation of GAL4 by the respective constructs was determined by measuring beta-glucuronidase activity. Reporter and effector gene plasmids were co-transfected into protoplasts isolated from *Arabidopsis* rosette leaves. Protoplasts were incubated in darkness for 20–22 h after transfection, and then GUS activity was measured. Data represented the mean ± SD of three replicates. T-test was used to analysis the significant defference (*P < 0.05; **p < 0.01). All tests were computed using SPSS(ver.17.0).

Previous studies demonstrated that the ability of exogenous bHLHs to induce endogenous gene expressions needed an efficient interaction with other endogenous MBW complex components such as MYB proteins. To decipher how FhGL3L and FhTT8L proteins networked with the *Arabidopsis* MYB regulators, AtPAP1, AtTT2 and AtGL1, which was specific for anthocyanin, proanthocyanidin biosynthesis and trichome formation, respectively, different combinations of effector and reporter constructs were designed and transiently expressed in *Arabidopsis* leaf protoplasts through PEG-mediated transfection method (Fig. 5A). Firstly, the tranactivation capacity was assessed using the GAL4-GUS system, and the results showed that only AtGL3 could activate the GUS expression among the four bHLH proteins, i.e. AtGL3, AtTT8, FhGL3L and FhTT8L (Fig. 5B). For the interaction analysis between FhGL3L and endogenous MYB regulators, FhGL3L-VP16 and GD-tagged AtPAP1/AtGL1/AtTT2 proteins were chosen as effectors. After the co-transfection, it can be observed that FhGL3L showed strong interactions with all the three AtMYBs (Fig. 5C). Because FhTT8L-VP16 showed low activation ability, three AtMYBs-VP16 (AtPAP1-VP16, AtGL1-VP16 and AtTT2-VP16) and GD-tagged FhTT8L were constructed as effectors to measure the interaction of FhTT8L. Unlike FhGL3L, it could only interact with AtPAP1 and AtTT2, no interaction was detected between FhTT8L and AtGL1 (Fig. 5D–F). Therefore, it can be concluded that the interaction specificities between *Freesia* bHLHs and *Arabidopsis* MYBs might be explained for their divergent functional roles.

### FhbHLHs-AtMYBs activated the promoters of flavonoid biosynthetic gene *AtDFR* and trichome formation related gene *AtGL2*

**Figure 6:**
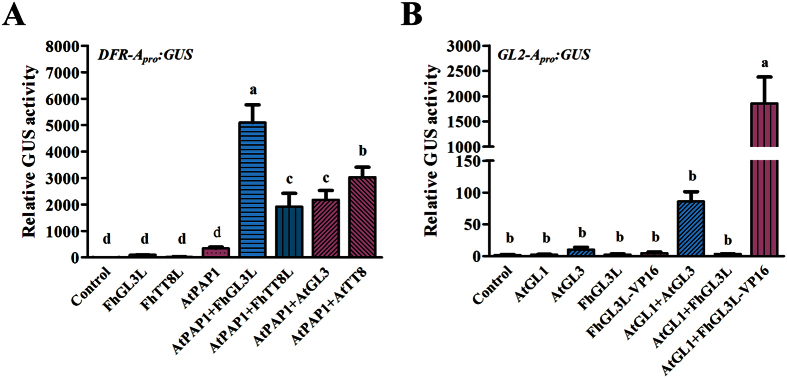
Promoter Activation of *AtDFR* and *AtGL2* on Protoplasts Transient Assays in *Arabidopsis.* (**A**) FhGL3L and FhTT8L interacted with AtPAP1 to activate *AtDFR*. Different construct combinations were transfected into *Arabidopsis* protoplasts. bHLHs from *Freesia* could activate *AtDFR* with AtPAP1. AtGL3 and AtTT8 were used as positive controls. (**B**) Transactivation capacity loss of FhGL3L resulted in the inhibition of trichome formation. Only FhGL3L tagged by VP16 co- transfected with AtGL1 could result in the activation of *AtGL2*, whereas FhGL3L could not. AtGL3 was used as control. Activation of GAL4 by the respective constructs was determined by measuring beta-glucuronidase activity. The plasmids were co-transfected into protoplasts isolated from *Arabidopsis* rosette leaves. Protoplasts were incubated in darkness for 20–22 h after transfection, and then GUS activity was measured. Data represented the mean ± SD of three replicates. Constructs were diagrammed at the bottom of the figure. One-way ANOVA was carried out to compare statistical differences (Ducan, p < 0.05). All tests were computed using SPSS(ver.17.0).

Using *Arabidopsis* leaf protoplasts transient expression system, the ability of FhbHLHs to regulate the *AtDFR* and *AtGL2* was tested. FhbHLHs were independently or co-transfected with the *Arabidopsis* MYBs together with the *pUC19-GUS* constructs, which contained the target promoters (*AtDFR* or *AtGL2*) driving the expression of the GUS reporter gene. Meanwhile, *Arabidopsis* endogenous MBW complex members, e.g. *AtPAP1*, *AtTT8*, *AtGL3* and *AtGL1*, were simultaneously introduced as positive controls. As expected, both FhGL3L and FhTT8L were able to activate the flavonoid biosynthetic gene *AtDFR* in combination with the MYB protein AtPAP1. Comparatively, the co-transfection of FhGL3L and AtPAP1 presented a significant stronger activating capacity compared with the FhTT8L and AtPAP1 combination (Fig. 6A), indicating that FhGL3L and FhTT8L might not perform the complete redundantly functions.

As mentioned above, AtGL3 was proved to be responsible for transcription activation in the AtGL1-AtGL3-AtTTG1 complex. To validate whether the lack of the transactivation ability of FhGL3L leads to the inhibition of the trichome formation, it was fused in-frame with a strong activation domain VP16. As expected, FhGL3L-VP16 could significantly activate the expression of the *AtGL2* when combined with AtGL1, whereas FhGL3L-AtGL1 had no effects and AtGL1/AtGL3 had weak effects (Fig. 6B).

## Discussion

MYBs and bHLHs that regulate the flavonoid biosynthetic pathway and trichome formation have been extensively described in many plant species^6^,^7^,^17^. Unlike MYB regulators, the effects of bHLH co-factors need further investigations in different evolutionary plant species^6^,^36^, especially in monocots. Different transcript profiles of bHLH genes have been found in dicot and monocot plant species. In maize, the bHLH R1/B1 genes (*R1*, *B1*, *Sn1*, *Lc1*, *Hopi1*), whose expression are tissue-specific, have been proved to determine the tissue distribution of pigments^9^. On the contrary, the transcription levels of three IIIf bHLHs, *LcbHLH1*, *LcbHLH2* and *LcbHLH3*, were not coordinated with anthocyanin accumulation in different tissues and during fruit development in litchi, despite *LcbHLH1* and *LcbHLH3* could enhance the anthocyanin accumulation in tobacco leaves when co-infiltrated with *LcMYB1*^37^. This is also consistent with what previously observed for *MdbHLH33*, *MdbHLH3*, and *VvMYC1*, which do follow neither the accumulation of anthocyanins nor the expression pattern of the MYB factor^38^,^39^.

In the present study, two IIIf subgroup bHLH regulator genes, named as *FhGL3L* and *FhTT8L*, were isolated from flowers of *Freesia hybrida*, which belonged to different phylogenetic clade that includes AtGL3 and AtTT8, respectively. We found that both of them expressed synergistically with flavonoid biosynthesis in plant tissues, whereas *FhTT8L* also showed positive correlation in anthocyanin accumulation during the flower developmental process. Comparatively, *FhGL3L* had a relative higher transcript level in most tested plant tissues and expressed higher in the early stages of the flower development, indicating that they might not perform completely redundant roles in flavonoid biosynthesis pathway and functioned divergently. When introduced into *Arabidopsis*, *FhGL3L* could significantly increase the anthocyanin accumulation through up-regulating *AtPAP1* and *AtTT8* as well as LBGs, and inhibit trichome formation on both stem and leaf lamina caused by the down-regulaiton of *AtGL2*. On the contrary, the overexpression of *FhTT8L* could not lead to obvious variation of trichome phenotype, but enhance the accumulation of both anthocyanins and proanthocynidins.

Since then, More than one bHLH factors have been found in most plants to regulate anthocyanin or proanthocyanidin biosynthesis, i.e., AtTT8, AtGL3, and AtEGL3 in *Arabidopsis*, PhJAF13 and PhAN1 in petunia, NtJAF13, NtAn1a and NtAn1b in tobacco, VvMYC1 and VvMYCA1 in grape, MdbHLH3 and MdbHLH33 in apple^22^,^36^,^38^, LcbHLH1 and LcbHLH3 in litchi^37^. Some of them have been proved to display clear functional differences evenwhen overexpressed, e.g. *AtGL3* and *AtEGL3* but not *AtTT8* could complement the *ttg1* mutants in *Arabidopsis*^40^, *PhJAF13* could not compensate for the loss of *PhAN1*, as *an1* mutants completely lack anthocyanins despite expressing *PhJAF13*^41^. Furthermore, a hierarchical regulation has also been observed between bHLH regulators. The bHLH factor *AtTT8* gene is activated by MBW complexes that include *AtTT8* itself or the bHLH factors *AtGL3* and *AtEGL3* in *Arabidopsis*^42^, and *AtEGL3* contributes more to *AtTT8* regulation than *AtGL3* even when *AtGL3* is overexpressed^15^. AN1 is directly involved in the activation of the biosynthetic genes, whereas JAF13 is involved in the regulation of AN1 transcription in Solanaceous plants^36^,^43^. In this study, a similar hierarchical regulation between *FhGL3L* and *FhTT8L* might also exist as *FhGL3L* expressed ahead of *FhTT8L* during the flower developmental process.

Although *FhGL3L* enhanced the anthocyanin accumulation when overexpressed in *Arabidopsis*, it could not activate the *AtDFR* promoter unless co-transfected with the endogenous anthocyanin biosynthesis related MYB protein, AtPAP1. In addition, *FhTT8L* was also failed to activate *AtDFR* promoter when not combined with AtPAP1. These results suggested that both IIIf subgroup bHLHs from *Freesia* do not regulate the biosynthesis of anthocyanins independent of endogenous MYB proteins. Indeed, we found that both FhGL3L and FhTT8L could interact with AtPAP1 and AtTT2, which was specific for anthocyanin and proanthocyanidin biosynthesis^15^,^16^, respectively.

There are increasing evidences that bHLH proteins fulfill essential roles in both dicot and monocot plants combined with MYB regulators. In *Arabidopsis*, the activation of the late biosynthesis genes, leading to the production of pro-anthocyanidins in seeds and anthocyanins in vegetative tissues, requires a ternary complex composed of MYB-bHLH-WD40 transcription factors^9^,^15^,^16^. Transgenic tobacco (*Nicotiana tabacum*) overexpressing a combination of either potato *StAN1* (MYB) with *StJAF13* (bHLH) or *StAN1* with *StbHLH1* showed deeper purple pigmentation with respect to AN1 alone^44^. In lily, the transient expression of *LhMYB6* and *LhMYB12* without *LhbHLH2* was not sufficient to activate the promoter of *LhDFR*, *LhCHSa* and *LhCHSb*. However, some studies showed that the overexpression of MYB regulator alone could efficiently induce anthocyanin accumulation, e.g. *LcMYB1* and *PeMYB2* from *Litchi* and *Phalaenopsis*^28^, repectively. bHLH regulators might also perform irreplaceable roles as the exogenous *LcMYB1* indeed induces the expression of the tobacco endogenous bHLH transcription factor, *NtAn1b*^45^.

**Figure 7:**
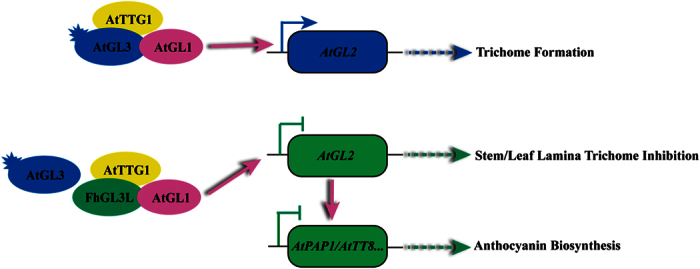
Schematic Diagram Representing the Roles of *FhGL3L* to Control the Anthocyanin Biosythesis and Trichome Formation in *Arabidopsis.* The regulatory proteins were represented by ellipse with different colors. Proteins with transactivation capacity are marked by sparks. Solid arrows represent direct transcriptional regulation, 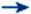 for activation and 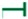 for repression, respectively, whereas the dotted arrow shows a series of increased concentration of genes leading to the biosynthesis of corresponding products.

In *Arabidopsis*, trichome formation on both stems and leaf lamina is controlled by a trimeric MBW complex composed of MYB (AtGL1)-bHLH (AtGL3/AtEGL3)-WDR (AtTTG1) proteins, which, in turn, activates the expression of *AtGL2*^7^, leading to the trichome formation. In the complex, only AtGL3 was found to have transactivation capacity (Figs 5B and 6B), which might act as the activator of the MBW complex. Because FhGL3L interacted with AtGL1 unlike FhTT8L (Fig. 5C–F), when they were introduced into *Arabidopsis*, only FhGL3L could compete AtGL1 with AtGL3 to form a new AtGL1-FhGL3L-AtTTG1 MBW complex to substitute the endogenous activator complex resulting in the expression inhibition of *AtGL2*. In addition, FhGL3L-VP16, a fused protein having a strong activation domain VP16, got the ability to activate the promoter of *AtGL2* when co-transfected with AtGL1 in *Arabidopsis* leaf protoplasts. These results suggested that bHLH proteins played determinant roles in the MBW complex responsible for trichome formation, and the loss of transactivation capacity of FhGL3L will explain for the glabrous phenotype of stems and leaf lamina in the transgenic lines (Fig. 7). Except for *AtGL3* and *AtEGL3*, *AtMYC1*and *AtTT8*, other members of the bHLH subgroup IIIf, have also been shown to affect trichome development^18^,^46^,^47^. *AtMYC1* mutants have less trichomes, compared with the wild type, indicating *AtMYC1* acts as a positive regulator of trichome initiation^47^. Maes *et al*.^18^ have demonstrated that *AtTT8* controlled trichome development on leaf margins in *Arabidopsis*, which might be the reason why the marginal trichomes retained in the *FhGL3L* transgenic lines^18^.

Previous studies showed that *AtGL2* was a versatile regulator involved in several bioprocess, such as epidermal cell-fate determination, mucilage biosynthesis, and seed oil production^7^,^10^,^12^,^48^,^49^,^50^,^51^. Our recent research also demonstrated that it was a transcriptional repressor, which functioned as a feed back loop to negatively control the anthocyanin biosynthesis in *Arabidopsis*, as the expression of LBGs was altered in a relative higher degree in *gl2-3* and *gl2-1D* mutants as well as components of the MBW complex involved in anthocyanin biosynthesis, such as *AtPAP1*, *AtPAP2*, *AtMYB113*, *AtMYB114* and *AtTT8*^19^. Therefore, it can be concluded that *AtGL2* played crucial roles to control the anthocyanin biosynthesis in *FhGL3L* transgenic lines, which was down-regulated, and in turn, directly activated the anthocyanin biosynthesis related transcription factor, e.g. *AtPAP1* and *AtTT8*, to regulate the expression of the LBGs leading to the hyper-accumulation of anthocyanins (Fig. 7). Furthermore, the interaction between FhbHLHs and endogenous AtPAP1 and AtTT2 was also contributable to the enhancement of anthocyanins and proanthocyanidins in transgenic plants.

## Materials and Methods

### Plant materials and growth conditions

Red River^®^, the world spreading cultivar of *Freesia hybrida* with red flowers, which was immigrated from Europe, was grown under greenhouse conditions to isolate IIIf-bHLH regulators involved in flavonoid biosynthesis and trichome formation. To investigate the spatio-temporal correlation between transcription profiles of bHLH genes and anthocyanins as well as proanthocyanidins accumulation patterns in flowers and different plant tissues, the flower developmental process was divided into five stages with increasing pigmentation intensities as described in our previous studies^29^,^30^, and 3 vegetative tissues, i.e. root, leaf and scape, and 5 flower tissues, i.e. torus, calyx, petal, stamen and pistil, were collected. All samples were immediately frozen in liquid nitrogen and stored at −80 °C until required.

*Arabidopsis* used for plant transformation and protoplast isolation was in the Columbia-0 (Col) ecotypic back-ground, and the wild type plants were grown in a growth chamber at 22 °C with 16 h/8 h (light/dark) photoperiod. About 5-week-old plants with several mature flowers in the main inflorescence were used for plant transformation. Leaves from plants which were about 3–4 weeks old were used for protoplast isolation. In order to study the flavonoids accumulation, transcription levels of exogenous and endogenous genes, seeds of wild type and transgenic plants were surface-sterilized, germinated and cultivated in 1/2 Murashige and Skoog (MS) medium (Sigma-Aldrich, http://www.sigmaaldrich.com) and anthocyanin induction medium (1/2 MS medium supplemented with 3% w/v sucrose), respectively. For the trichomes observation, seedlings grown on the medium were transplanted into soil pots.

### DNA or RNA Extraction and cDNA Synthesis

DNA was extracted from *Freesia* flowers using NuClean Plant Genomic DNA Kit (CWBIO) according to the manufacturer’s instruction. RNA was extracted from different samples of *Freesia* or *Arabidopsis* using RNAiso Plus (TaKaRa) kit. Contaminating DNA was removed from RNA preparations with DNaseI (TaKaRa). cDNA was synthesized from total RNA (1 μg) using Oligo d(T)15 primers together with M-MLV Reverse Transcriptase (Promega) following the manufacturer’s instructions.

### Gene cloning and sequence analysis

To isolate the candidate bHLH genes, *in situ* tblastn screen of *Freesia* transcriptomic database, including transcripts from five flower developmental stages and five flower tissues aforementioned, was conducted using *AtGL3* and *AtTT8* as probe baits. Sequences obtained were subjected to manual blastx search of National Center for Biotechnology Information, and the best hits were defined as candidate genes. As the two IIIf-bHLH regulator genes, named as FhGL3L and FhTT8L which showed identities to *Arabidopsis GL3* and *TT8*, respectively, were predicted to have intact ORFs (open reading frame), specific primers were designed (Table S2) to amplify the full length cDNA sequences from blossoming flowers. To ascertain the genomic structures of two bHLHs, combinations of primers were designed (Table S2) to amplify their genomic sequences. PCR products of appropriate length were cloned into pGEM-Teasy vector (Promega) and then transformed into *E.coli* JM109 competent cells before sequencing.

Multiple sequence alignment was performed using Clustal Omega, MIR, WD40/AD, bHLH, and ACT-like regions were highlighted with different colors. For phylogenetic analysis, the full-length amino acid sequences of FhTT8L and FhGL3L protein and their homologs in other plant species were aligned with the Clustal Omega using default parameters (http://www.ebi.ac.uk/Tools/msa/clustalo/), and then the alignments were subjected to MEGA version 6 to generate a neighbor-joining tree with bootstrapping (1,000 replicates) analysis and handling gaps with pair wise deletion.

### Plant transformation

Before transformation, *FhGL3L* and *FhTT8L* were cloned into pBI121 vector harboring the *CaMV* 35S constitutive promoter and confirmed by sequencing. About 5-week old *Arabidopsis* plants with a few mature flowers on the main stems were transformed by the floral dip method^52^. T1 seeds were selected on 1/2 MS medium containing 50 mg L^−1^ kanamycin then transferred to soil to set T2 seeds. After 1 week of culture on anthocyanin gene induction media^53^, three independent transgenic lines were subjected to further analysis. Elevated expression of *FhGL3L* and *FhTT8L* in transgenic plants was confirmed by RT-PCR.

### Anthocyanin and proanthocyanidin analysis

300mg of different *Freesia* samples and 7-day old wild type or transgenic *Arabidopsis* seedlings grown on anthocyanin induction medium were ground into fine powder in liquid nitrogen. Then the powdered samples were used for both anthocyanin and proanthocyanidin analysis. Total anthocyanin content was estimated as described by Pandey^54^. Briefly, the powdered samples were extracted with 1% acidic methanol for 18 h at room temperature followed by a centrifugation at 12000 rpm for 1 min. After centrifugation, 400 μl of supernatant was collected and mixed with 600 μl of acidic methanol. Absorbance of the sample was recorded at 530 nm (A530) and 657 nm (A657). The anthocyanin content was quantified as (A530–0.25 × A657) g-1 fresh weight (FW).

The amount of proanthocyanidin was determined by the DMACA-HCl method as reported previously^55^. Briefly, powdered samples were extracted in microcentrifuge tubes with 70%(V/V) aqueous acetone solution containing 0.1%(W/V) ascorbic acid at 4 °C. The supernatants were mixed with diethyl ether and left at −20 °C in the dark for the separation of two phases. Then the lower phase used for assay of soluble proanthocyanidin was mixed with methanol and DMACA reagent, left at room temperature for 20 min and the absorbance at 640 nm was measured. The proanthocyanidin content was quantified as A640 g-1 fresh weight (FW).

### Real time qPCR analysis

To investigate the gene expression profiles, Real-time qPCR was carried out with ABI StepOne Plus Real-Time PCR System (USA) and SYBR Master Mix (TOYOBO). The specific qRT-PCR primers of *FhGL3L* and *FhTT8L* were designed using Primer Premier 5 and listed in Supplementary Table S2, Primers for flavonoid biosynthetic genes and MBW activator complex component genes have been described previously^4^,^15^,^56^,^57^ as well as trichome formation related MBW complex component genes^19^,^58^. PCR parameters were set as previously reported^30^ with a negative control using water as template, and the *18S rRNA* and *Actin* genes were used as internal control for *Freesia* and *Arabidopsis*, respectively. Data of the gene expression was calculated with formula 2^−ΔΔCт ^59, and all biological replicates were measured in triplicate.

### Plasmid DNA preparation used in *Arabidopsis* leaf protoplast transfection assay

To generate GD (Gal4 DNA binding domain)-tagged constructs for leaf protoplast transfection, corresponding sequences were cloned in frame with an N-terminal GD tag into the *pUC19* vector under the control of the double 35S promoter of *CaMV*, and terminated by the 3′ UTR (untranslated region) derived from nopaline synthetase gene^19^.

To investigate the transregulation properties of FhGL3L and FhTT8L, GD-tagged constructs harboring the full length, N-terminal region, i.e. FhGL3LN and FhTT8LN, the truncated protein including MIR and WD/AD domains, and C-terminal region, i.e. FhGL3LC and FhTT8LC, the truncated protein including bHLH and ACT-like domains, respectively, of the two FhbHLHs were generated.

FhGL3L-VP16 and GD-tagged AtPAP1/AtGL1/AtTT2 proteins were chose as effectors to examine the interaction of FhGL3L with *Arabidopsis* MYB proteins, whereas AtMYBs-VP16 (AtPAP1-VP16, AtGL1-VP16 and AtTT2-VP16) and GD-tagged FhTT8L were constructed as effectors for FhTT8L interaction analysis, because FhTT8L-VP16 was proved to have low activation transcription efficiency.

To verify whether FhbHLHs-AtMYBs could activate the promoters of flavonoids biosynthesis and trichome formation related genes, a 1277 bp *AtDFR* promoter (Accession number: AT5G42800) from *Arabidopsis thaliana* was amplified, and the *AtDFR-pro:GUS* construct was generated. Meanwhile, the *AtGL2-pro:GUS* vector was shown in our previous studies^19^. Both two constructs were used as reporter plasmids, whereas recombinant *pUC19* vectors carrying *FhGL3L*, *FhGL3L-VP16*, *FhTT8L*, *AtPAP1*, *AtGL3* and *AtGL1* were used as effectors.

All the effector and reporter plasmids were prepared using the EndoFree Plasmid Maxi Kit (Qiagen) following the manufacturer’s instructions.

### Protoplast isolation, transfection and GUS activity assay

Protoplast isolation, transfection and GUS activity assays were performed as described previously^58^,^60^. Briefly, protoplasts were isolated from rosette leaves collected from 3 to 4-week-old Col wild type plants. Effector plasmids encoding GD alone were transfected with different combinations of effector and reporter plasmids into protoplasts. The protoplasts were incubated at room temperature for 20–22 h under darkness before GUS activities were measured using a SynergyTM HT microplate reader (BioTEK, www.biotek.com).

## Additional Information

**How to cite this article**: Li, Y. *et al*. Two IIIf Clade-bHLHs from *Freesia hybrida* Play Divergent Roles in Flavonoid Biosynthesis and Trichome Formation when Ectopically Expressed in *Arabidopsis*. *Sci. Rep.* **6**, 30514; doi: 10.1038/srep30514 (2016).

## Supplementary Material

Supplementary Information
