# Supplementary material for: Two IIIf Clade-bHLHs from Freesia hybrida Play Divergent Roles in Flavonoid Biosynthesis and Trichome Formation when Ectopically Expressed in Arabidopsis
Source: Sci Rep. 2016 Jul 28;6:30514. doi: 10.1038/srep30514 (PMC4964595; doi:10.1038/srep30514)
Supplement: Supplementary Information [file srep30514-s1.pdf]

Two IIIf Clade-bHLHs from *Freesia hybrida* Play Divergent Roles in Flavonoid Biosynthesis and Trichome Formation when Ectopically Expressed in *Arabidopsis*

Yueqing Li[1, 2], Xiaotong Shan[1, 2], Ruifang Gao[1, 2], Song Yang[1, 2], Shucai Wang[1, 2], Xiang Gao[1, 2*], Li Wang[1, 2*]

[1] Key Laboratory of Molecular Epigenetics of MOE, Changchun, China

[2] Institute of Genetics and Cytology, Northeast Normal University, Changchun, China

*To whom correspondence should be addressed. E-mail address: gaoxiang424@163.com; wanglee57@163.com
Tel.:+86 431 85099360;

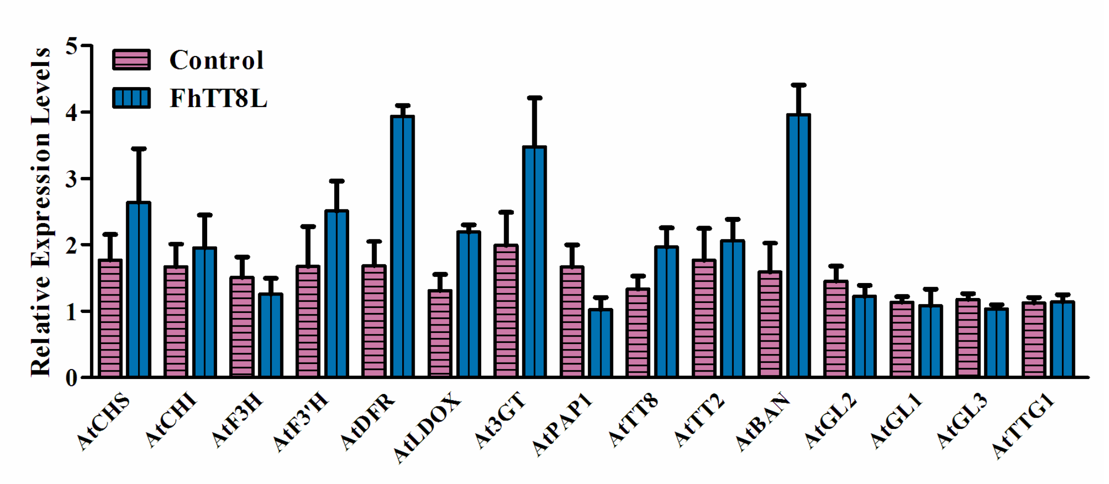

Figure S1. Expressional Analysis of Genes involved in Flavonoid Biosynthesis and Trichome Formation in the Wild Type and FhTT8L Transgenic Protoplasts.

Table S1. Information of bHLH Regulatory Genes

Candidate transcripts	Protein sequence length	Function Prediction	Top *Arabidopsis* BLAST match	Top BLAST match excluding *Arabidopsis*	Homology (%)
**FhTT8L**	**697 residues**	**TT8-like**	**NP_192720.2 *TT8*<br>*Arabidopsis thaliana***	**XP_006479738.1 *TT8*<br>*Citrus sinensis***	**57[a],57[b]**
**FhGL3L**	**686 residues**	**EGL3,GL3-like**	**NP_192720.2 *TT8*<br>*Arabidopsis thaliana***	**XP_008806796.1 *GL3*<br>*Phoenix dactylifera***	**40[a],63[b]**

a% Similarity to Arabidopsis.
b% Similarity to other plant sequence

Table S2: Primers Used in the Study

		Forward(5'-3')	Reverse(5'-3')
cDNAs	FhTT8L	GTAACATTTCCTTAAACTATA	CATGATGGGATAAGGAATCAGTGAA
	FhGL3L	TTTTGCTGGAAAGATTGGAT	TGTGCCACTAACAAAATGTATCTTC
Genomic sequences	TT8-1	ACAAATGGCGTCGCAGCAGT	TTGAACGTGGAAATTAAAGG
	TT8-2	ATGGCGTCGCAGCAGTCG	CTAGTATTGGGAGAAGATATGGTG
	TT8-3	TTCCACAAAGTGAACCAAG	ATCAGGAGAGCCAAGTCGG
	TT8-4	GACCAGAGCAGTACCCTAAC	CTTCCTCTTGTCCACGGC
	GL3-1	TGGTAGCACATCTCAGAAAC	GGAAGTTGTTACTTGTTGTATGAG
	GL3-2	ATGTGTCACTCAATGGAAGG	CTGTTGGCATCTTCATTATT
Generation of constructs	FhTT8L	TCCCCCGGGATGGCGTCGCAGCAGTCG	TCCCCCGGGAACGTGGAAATTAAAGGTTTT
	FhGL3L	CGGGATCCATGTGTCACTCAATGGAAGG	TCCCCCGGGTGCCACTAACAAATGTATCT
Transient Luciferase Assay	FhTT8LN	ATGGCGTCGCAGCAGTCGAG	CTACCATCTAGAGAATGCCGAT
	FhTT8LC	ATGCACCTATACCATTCCC	CTAGTATTGGGAGAAGATAT
	FhGL3LN	ATGTGTCACTCAATGGAAGG	TCACCAAATTGTGAAGCTGGAT
	FhGL3LC	ATGGAAATTTTTTATCATGGAA	TCAACATATGCCAACAACTCT
	AtPAP1	ATGGAGGGTTCGTCCAAAG	CTAATCAAATTTCACAGTCTCTCC
	AtTT2	ATGGGAAAGAGAGCAACTACT	TCAACAAGTGAAGTCTCGGAG
	AtGL1	ATGAGAATAAGGAGAAGAGAT	CTATTGCCGAGGAGCTTGTGG
	AtTT8	ATGGATGAATCAAGTATTATTC	CTATAGATTAGTATCATGTATTATG
	AtGL3	ATGGCTACCGGACAAAACAG	TCAACAGATCCATGCAACCCT
	VP16	ATGGTCTTTGATAGCTAAAAG	CTAAAGGCAGTACTCAACATC
Promoter	ProAtDFR	GAGATTGGCACCACCTTCGCCTC	TTTTGTGGTTATATGATAGATTGTGCT
	ProAtGL2	TTAAAAGGGATCGTCGTCAC	TTTTTCTTCTTAATATTCGATTTT
qRT-PCR	FhTT8	CTCTTGAAACGCAGCGACAT	TTTGCACTCTTGGCTAGGAT
	FhGL3	GAAAGACCACAGAAAGCAGC	TTGTAGTATCCGTCACCCCA
	AtCHS	GGCAAAGAAGCGGCAGTGAAG	CGGAAGGACGGAGACCAAGAAG
	AtCHI	CTCTCTTACGGTTGCGTTTTCG	CACCGTTCTTCCCGATGATAGA
	AtF3H	GACCAAGTCGGTGGATTACAAGC	TCCTTCAACAGGCTGAACCG
	AtF3'H	TTCCTTACCTTCAGGCGGTTATC	CGAGAGTGGTGTTGGTGGATG
	AtDFR	CTTTGTTCGTGCCACCGTTCG	TCCTTCCTCAGATAAATCAGCCTTCC
	AtLDOX	GTTTGCAGCTTTTCTACGAGG	TGAGCAAAAGTCCGTGGAGG
	At3GT	TGTCAGATCGTTTTGGTTCC	GATTCTTCCTCACTTTCTCAC
	AtBAN	AACAACTAAATCTCTATCTCTGTA	GAATGAGACCAAAGACTCATATAC
	AtPAP1	CTTGGAACAGAGCTTTTGACCG	CGTGAAAACCTTGTCGAACTCTTC
	AtTT8	GGCGGTTCAATCTGTGGAC	CTGTTGGCTCCTCTCTAACG
	AtGL3	TCGGTTCGTTTGGTAATGAGG	GCTTGCAATTGACGGTTAAGC
	AtTT2	CCGCAAAAGACTTCCCAAAA	GCACCTAATCGCCTTTGTACGT
	AtTTG1	TCGATATTCGTTCGCCGACT	GCCTGTGTATCATCACCACCAG
	AtGL1	AATGGCAACTACTAATGATCCAAG	CTAAAGGCAGTACTCAACATCACCA
	AtGL2	AGGCTATTCAAGAACGGCACGAGA	AGCTTATCGAGCTCGGCTTTCAGT
	ACTIN	GCTGAGAGATTCAGATGCCCA	GTGGATTCCAGCAGCTTCCAT
